# Views of nursing staff on computerized dementia screening: A validation and pilot study in a general hospital

**DOI:** 10.1007/s00391-019-01633-0

**Published:** 2019-10-22

**Authors:** Stelios Zygouris, Mara Gkioka, Despoina Moraitou, Birgit Teichmann, Thrasyvoulos Tsiatsos, Sotirios Papagianopoulos, Magda Tsolaki

**Affiliations:** 1 School of Medicine, Aristotle University of Thessaloniki, Thessaloniki, Greece; 2 Network Aging Research, Heidelberg University, Heidelberg, Germany; 3 School of Psychology, Aristotle University of Thessaloniki, Thessaloniki, Greece; 4 School of Informatics, Aristotle University of Thessaloniki, Thessaloniki, Greece; 5 Greek Association of Alzheimer’s Disease and Related Disorders, Thessaloniki, Greece

**Keywords:** Cognitive disorders, Questionnaire, Attitudes, Reliability, New technologies, Kognitive Störung, Fragebogen, Einstellungen, Reliabilität, Neue Technologien

## Abstract

**Background:**

Cognitive disorders such as dementia are common among older adults admitted to general hospitals. They can complicate treatment leading to longer hospitalization and worse outcomes. They often remain underdiagnosed as the busy routine of the hospital does not enable efficient screening and available screening instruments are not suitable for the hospital environment. Computerized cognitive testing (CCT) has been proposed as an efficient screening method as it can be employed by nonspecialists, such as nurses while featuring automatic scoring and interpretation of results.

**Objective:**

This study validated a newly developed questionnaire for measuring the attitudes of Greek nurses towards computerized dementia screening.

**Material and methods:**

The questionnaire was validated in a sample of 212 undergraduate psychology students and subsequently administered to a sample of 19 nurses working in a general hospital. Reliability of the questionnaire was calculated using Cronbach’s alpha (= 0.762). Factor analysis revealed the existence of a single factor (acceptability-feasibility) that accounted for 33.73% of variance with an eigenvalue of 3.036.

**Results:**

The total score of all the items loading on the single factor (acceptability-feasibility) was calculated. Scores ranged between 10 and 40 with the average score for the validation group being 29.33 (SD = 4.89) and the average score for the nurses’ group being 29.50 (SD = 3.20).

**Discussion:**

The questionnaire has acceptable reliability. Results indicate that acceptability-feasibility is high in both groups and there were no statistically significant differences between the two groups.

**Electronic supplementary material:**

The online version of this article (10.1007/s00391-019-01633-0) contains supplementary material, which is available to authorized users.

## Zusammenfassung

**Hintergrund:**

Kognitive Störungen wie Demenz sind eine häufige Nebendiagnose von älteren Menschen in Allgemeinkrankenhäusern und können zu einer verlängerten Verweildauer und zu schlechteren Therapieergebnissen führen. Sie werden oft nicht diagnostiziert, da im Krankenhausalltag keine effiziente Untersuchung möglich ist und die verfügbaren Untersuchungsinstrumente nicht für diese Umgebung geeignet sind. Computergestützte kognitive Tests (CCT) stellen eine effiziente Screening-Methode dar, da sie von Nichtfachleuten wie Pflegepersonal angewendet werden können und eine automatische Bewertung und Interpretation der Ergebnisse ermöglichen.

**Ziele:**

In dieser Studie wurde ein neu entwickelter Fragebogen zur Messung der Einstellungen griechischen Pflegepersonals zum computergestützten Demenz-Screening validiert.

**Methoden:**

Der Fragebogen wurde an einer Stichprobe von Psychologiestudenten (*n* = 212) validiert und anschließend an Pflegekräften (*n* = 19) in einem Allgemeinkrankenhaus angewandt. Aus der Faktorenanalyse ergab sich eine Lösung mit einem einzelnen Faktor (Akzeptanz – Machbarkeit), der 33,73 % der Varianz mit einem Eigenwert von 3,036 ausmacht.

**Ergebnisse:**

Es wurde die Summe aller Elemente, die auf diesen Faktor geladen wurden, berechnet. Der Score lag zwischen 10 und 40, wobei der mittlere Score für die Validierungsgruppe *M* = 29,33 (*SD* = 4,89) und für die Gruppe der Pflegekräfte *M* = 29,50 (*SD* = 3,20) betrug.

**Diskussion:**

Die Ergebnisse zeigen, dass die Akzeptanz – Machbarkeit in beiden Gruppen hoch ist und es keine statistisch signifikanten Unterschiede zwischen den beiden Gruppen gibt.

Despite recent medical advances, dementia remains underdiagnosed in older adults admitted to general hospitals. High workload of the hospital staff in combination with lack of appropriate screening tools further exacerbate this issue. Computerized cognitive testing (CCT) has been proposed as a solution as it can be administered by nonspecialists such as nurses while featuring automatic scoring and interpretation of results. At the same time, it has not been integrated in healthcare systems and the attitudes of healthcare personnel as well as the barriers to its implementation have not been studied.

## Background

### Dementia and mild cognitive impairment in general hospitals

Cognitive disorders such as dementia and mild cognitive impairment (MCI) are common in older adults. As this age group makes frequent use of healthcare services such as general hospitals these disorders can affect all aspects of therapy and care from communication with hospital staff to adherence to therapy. People with dementia (PwD) are more likely to stay longer in hospital and the clinical outcome is predominantly negative [1]. Up to 75% of people with dementia admitted to an emergency department exhibit behavioral and psychological symptoms of dementia (BPSD) [2], which include agitation, aggression, mood disorders, wandering and sleep disorders [3]. The occurrence of BPSD causes difficulties in management and significant distress to family and professional caregivers and is often aggravated during an acute hospital admission [4–6]. At the same time PwD experience difficulties in many aspects of the communication with hospital staff [7]. This can aggravate the psychosocial problems and trigger more BPSD, such as aggression [8]. Additionally, the hospital environment can be disorientating and stressful for PwD further exacerbating BPSD [9]. Early detection of dementia and management of BPSD can lead to reduced distress for nursing staff and patients and improved health outcomes for patients [10, 11]. Unfortunately, cognitive disorders often remain undiagnosed in the busy environment of a general hospital. Inadequate staff training and the lack of easy to use and reliable screening instruments further exacerbate this issue. Hospital staff usually have inadequate training and skills for caring for PwD and such a deficit in skills and training not only causes BPSD in PwD but also high levels of stress to hospital staff [12–14]. It is known that the quality of care in general hospitals is inadequate for the needs of PwD [15, 16], thus staff training in dementia-related issues is a pressing need [14]. At the same time there is an unmet need for quick easy to use screening instruments for cognitive disorders that can be administered by nonspecialists in general hospitals [17]. As national dementia plans are being implemented one of the core goals in many countries is dementia training for hospital staff [18, 19]. Thus, recently a large number of staff training programs have been implemented [9].

### Computerized cognitive testing

Computerized cognitive testing (CCT) is often presented as an easy way to detect and evaluate cognitive disorders [20, 21]. Automatic recording and scoring of responses, standardization of administration and reduction of examiner bias, high accuracy in measuring variables such as reaction time and the ability to tailor the difficulty level to the ability level of the examinee make CCT suitable for use in various clinical environments [21]. Following the need to screen an ever-increasing number of older adults, computerized tests have shifted the focus towards screening by adopting certain characteristics. The short administration time (10–20 min), ability to be administered by nonspecialists such as nurses or healthcare assistants and the use of portable commercially available devices such as tablet personal computers (PC) make them ideal for screening in busy healthcare services from primary care to general hospitals [17]. At the same time the implementation of CCT in healthcare and the attitudes of healthcare professionals towards them have not been studied in detail. Some research efforts have focused on primary care and results have been encouraging [22–24]. Additionally, the attitudes of patients towards CCT appear to be positive despite suggestions for improvements of existing tests [25]; however, the implementation of CCT in general hospitals and the attitudes of hospital personnel towards it has not been studied. Studies assessing attitudes of hospital personnel towards technological solutions have focused on organizational aspects and digital patient records [26–28] and not on computerized dementia screening. Implementation studies in hospitals and assessment of the attitude of healthcare personnel towards CCT is needed in order to assess whether its proposed advantages lead to actual improvements in healthcare.

## Methods

### Study design

This study was comprised of a validation study of a new psychometric tool and a cross-sectional survey to assess the attitude of registered Greek nurses towards computerized dementia screening instruments in general hospitals. A new questionnaire was created for this study as other suitable questionnaires were not available. The questionnaire was initially validated on a sample of psychology students before being administered to a sample of nurses from a general hospital.

### Participants

The sample of this study comprised two groups. The sample used in the validation of the questionnaire included 212 undergraduate psychology students from the School of Psychology of the Aristotle University of Thessaloniki. The third-year students attending the old age psychology course were recruited as a convenience sample. Students were informed about the purpose of the study and invited to complete the questionnaire. Out of 220 students who were invited, 212 chose to complete the questionnaire and provided consent for participation in the study. The questionnaire was also administered to a sample of 19 nurses working at the Papanikolaou General Hospital of Thessaloniki in order to collect preliminary data on the attitude of Greek nurses towards computerized dementia screening instruments in general hospitals. These nurses were participating in a short dementia training program and were administered this questionnaire along with other scales as part of an assessment of the knowledge of dementia. Nurses were informed that they could voluntarily complete the questionnaire as part of a study. All invited nurses consented to complete the questionnaire and participate in the study. Concerning the age of participating nurses, 10.5% were 26–35 years old, 15.8% were 36–45 years old, 63.2% were 46–55 years old and 10.5% were 56–65 years old. Concerning work experience of participating nurses, 5.3% had 6–10 years of work experience, 10.5% had 11–15 years of work experience and 84.2% had over 15 years of work experience. All participating nurses except one had completed postsecondary education of some form.

### Tools

A literature search revealed the lack of any similar questionnaires. An 8‑item questionnaire for measuring the attitudes of nurses towards the use of CCT was developed by the authors for this study (see supplementary material). The questionnaire comprises four items scored on a Likert scale, one item scored on a binary scale and three items scored on an ordinal scale. The length of the questionnaire and the structure of items were selected after a pilot trial and assessment of feedback from nurses and healthcare experts to ensure good face validity and acceptability. A researcher specializing in neuropsychology of old age and a coordinator of dementia training programs for nurses also provided feedback and participated in the creation of the final version of the questionnaire. The overall aim was to create a questionnaire that is quick to administer and focuses on the acceptability of computerized screening tests and the willingness of nurses to use them. Apart from the four core questions assessing acceptability, three further questions were added to collect data on practical aspects and barriers to implementation of computerized screening in general hospitals. A question assessing familiarity with computerized screening instruments was also included. The questionnaire was initially validated on a sample of psychology students. An exploratory factor analysis was conducted on items with Likert scale scoring to test its structural validity and it revealed a single factor (acceptability-feasibility) accounting for 33.73% of variance with an eigenvalue of 3.036. Factor analysis component matrix is presented in Table 1. A scree plot supported the existence of a single factor. The Kaiser-Meyer-Oklin (KMO) index was found to be 0.754 and Bartlet’s test of sphericity was found to be statistically significant (χ^2^ (36) = 347.491, *P* < 0.001). Reliability of the questionnaire was calculated using Cronbach’s alpha and it was acceptable (alpha = 0.762).

**Table 1:** Factor analysis component matrix

Component matrix	
	Acceptability-feasibility^a^
Question 2d Embedded diagnostic algorithm	0.730
Question 2c Automated scoring	0.700
Question 2e Information for further referral	0.689
Question 2a Fast administration	0.671
Question 2b Use by nonspecialists	0.597
Question 5 Willingness of nurses	0.543
Question 7 Willingness or relatives	0.494
Question 6 Willingness of older adults	0.439

All questions were scored in a 5-point Likert scale (Not at all/A little/So-so/A lot/Very much)

Question 2: How desirable do you consider the following characteristics of computerized dementia screening tests? This question comprised six subitems. Question 2a: brief administration, question 2b: ability to be administered by most members of staff (nurses/doctors/psychologists/other healthcare personnel), question 2c: automated administration and scoring, question 2d: embedded diagnostic algorithm, question 2e: provision of information for care/referral after the end of the examination, question 2f: other (description added by the examinee). Question 5: How interested you would be in using tests that are administered to relatives of patients and to patients with a high level of functionality, and can be used autonomously by the examinee without your participation in the examination (self-administered tests)? Question 6: Do you believe that older adults without serious memory issues and with good functionality would be interested in using a self-administered cognitive assessment test? Question 7: Do you believe that relatives of older adult patients will be interested in using a self-administered computerized questionnaire where they will evaluate the everyday functionality of the patient before admission to hospital and receive information about the probability of the patient suffering from dementia?

^a^Numbers represent factor loadings, which are the correlations between the variable and the factor

## Results

### Frequencies analysis

The total score of all the items loading on the single factor (acceptability-feasibility) was calculated. The minimum value for that score is 8 while the maximum value is 40. Frequencies were also calculated for those items that were not included in the factor analysis because they were not scored on a Likert scale.

### Validation sample

In the validation sample the total score had an average value of 29.33 (SD = 4.89). Frequencies for each answer on dichotomous questions/subitems are presented in Table 2 while frequencies for ordinal questions are presented in Table 3. The most common answer to question 3 (how much time could you devote to training in the use of computerized dementia screening tests?) was 1 week. The most common answer to question 4 (how much time could you devote during your shift to examine an older adult with a computerized dementia screening test?) was 20–40 min.

**Table 2:** Frequency analysis for dichotomous questions and subitems—validation sample

	Yes (%)	No (%)
Question 1	23	77
Question 8a	91.2	8.8
Question 8b	63.4	36.6
Question 8c	74.1	25.9
Question 8d	63.9	36.1
Question 8e	64.4	35.6
Question 8f	31.2	68.8

Question 1: Are you aware that there are screening tests for dementia that are administered through a computer or table device?

Question 8: Which factors do you believe are hampering the integration of computerized dementia screening tests in your hospital? This is comprised of six subitems. Question 8a: cost of equipment, question 8b: cost of software, question 8c: insufficient training, question 8d: lack of a plan for the integration of computerized screening tests in the daily routine of the hospital, question 8e: time needed for staff training, question 8 f: time needed for test administration

**Table 3:** Frequencies analysis for ordinal questions—validation sample

Question 3	Less than 4 h	4–8 h	1–2 days	3–4 days	1 week
–	11.1%	12%	20.2%	14.9%	41.8%
Question 4	Less than 5 min	5–10 min	10–20 min	20–40 min	40–60 min
–	0.5%	3.4%	24.5%	38.2%	33.3%

Question 3: How much time could you devote to training in the use of computerized dementia screening tests?

Question 4: How much time could you devote during your shift to examine an older adult with a computerized dementia screening test?

### Nursing staff sample

In the nursing staff sample the total score had an average value of 29.50 (*SD* = 3.20). Frequencies for each answer on dichotomous questions/subitems are presented in Table 4 while frequencies for ordinal questions are presented in Table 5. The most common answer to question 3 (how much time could you devote to training in the use of computerized dementia screening tests?) was 4–8 h. The most common answers to question 4 (how much time could you devote during your shift to examine an older adult with a computerized dementia screening test?) were 5–10 min and 10–20 min.

**Table 4:** Frequencies analysis for dichotomous questions and subitems—nursing staff sample

	Yes (%)	No (%)
Question 1	36.8	63.2
Question 8a	68.4	31.6
Question 8b	15.8	84.2
Question 8c	42.1	57.9
Question 8d	63.2	36.8
Question 8e	52.6	47.4
Question 8f	47.4	52.6

Question 1: Are you aware that there are screening tests for dementia that are administered through a computer or table device?

Question 8: Which factors do you believe are hampering the integration of computerized dementia screening tests in your hospital? This question comprised six subitems. Question 8a: cost of equipment, question 8b: cost of software, question 8c: insufficient training, question 8d: lack of a plan for the integration of computerized screening tests in the daily routine of the hospital, question 8e: time needed for staff training, question 8 f: time needed for test administration

**Table 5:** Frequencies analysis for ordinal questions—nursing staff sample

Question 3	Less than 4 h	4–8 h	1–2 days	3–4 days	1 week
–	5.3%	31.6%	26.3%	21.1%	15.8%
Question 4	Less than 5 min	5–10 min	10–20 min	20–40 min	40–60 min
–	0%	31.6%	31.6%	26.3%	10.5%

Question 3: How much time could you devote to training in the use of computerized dementia screening tests?

Question 4: How much time could you devote during your shift to examine an older adult with a computerized dementia screening test?

## Discussion

This is the first study that created a questionnaire for measuring the attitude of Greek nurses towards computerized cognitive screening. The results of this study should be considered within the context of everyday clinical practice in a general hospital and the information that is currently available for the implementation of CCT in healthcare. Studies in primary care settings have demonstrated that even when patients self-administer the screening test the length of the report and the time needed to interpret it can be a barrier to CCT use [23, 24]. A critical view of new technology and its potential benefits and limitations is necessary to ensure that CCT leads to actual improvements in quality of care and health outcomes [17, 21, 22]. At the same time lessons learned from research on the acceptance of other forms of medical information and communication technology (ICT), such as electronic medical records (EMR), by nurses should be taken into consideration. The benefits of new technologies will not be fully realized if such technologies are underutilized after implementation [29]. Acceptance and utilization of new technological instruments depend on how they are perceived by healthcare personnel [30]. Healthcare professionals accept change at different rates and individual anxieties and reservations have to be accounted for and discussed [29]. Anxiety and fear about their usefulness and ease of use can lead to underutilization [29, 30]. Computer literacy and familiarity with new technologies can have a positive effect on the attitude of nurses and their acceptance of medical ICT, therefore training programs should focus on enhancing ICT skills [29, 31]. The ICT skills training programs that run on a regular basis and feature follow-up training have been successful in increasing computer usage even in nurses with no computer familiarity [29]. Finally, organizational change is needed to support the utilization of novel medical ICT systems. The working environment should promote the use of ICT and provide the necessary support in terms of time and equipment [29]. Hospitals could also recruit, internally or externally, nurses who are familiar with ICT and optimistic about its usefulness in the hospital environment to act as champions of ICT by helping their colleagues in utilizing newly adopted ICT tools [32].

## Study limitations

The limitations of this study include that the validation of the questionnaire was conducted with psychology students and not with nurses, the lack of established measures for measuring similar attitudes (as none such measure is available) and the small number of nurses included in the study.

## Conclusion

The questionnaire that was created and validated is brief and has acceptable psychometric properties. The existence of a single factor (acceptability-feasibility) is in line with what was expected as the main purpose of the questionnaire is to evaluate willingness to use computerized cognitive screening instruments. The items that were not loading on the factor comprise ordinal questions designed to offer further data on implementation of computerized screening tests in a hospital environment.

A comparison of the average score of psychology students (validation sample) and nurses revealed no statistically significant differences thus indicating that the acceptability of computerized dementia screening remains high in both groups. At the same time there were differences between groups in the responses to the ordinal questions designed to offer further data on computerized screening implementation. Nurses were more aware about the existence of computerized screening tests and could devote less time for training and test administration. Concerning barriers to the implementation of computerized screening, cost of equipment was considered a barrier by a large percentage of participants in both groups while cost of software was considered a barrier mainly by the students group. Insufficient training was considered a barrier mainly by the students group while lack of a plan for the integration of computerized screening tests in the daily routine of the hospital was considered a barrier by a large percentage of participants in both groups. Time needed for staff training was considered a barrier by a large percentage of participants in both groups while time needed for test administration was not considered a barrier by the majority of the student group while it was considered a barrier by half of the nursing staff sample.

The results of the study provide moderate support for the utility of the questionnaire and indicate that it may be a valid tool for measuring attitudes of Greek healthcare personnel towards computerized testing. At the same time the results from the nursing staff sample indicate that the overall attitude of nurses towards computerized dementia screening is positive despite the existence of certain barriers towards its implementation in general hospitals. Further studies should be conducted to address the limitations of the current study. This could include further validation of the questionnaire in a large sample of nurses and an observational study on the application of computerized screening by nurses. Additionally, implementation studies in Greek general hospitals are necessary to establish the effect of CCT in older patients’ health outcomes.

## Caption Electronic Supplementary Material

Questionnaire for the use of new technologies for timely detection of dementia

## References

[1] Moyle W (2008). Best practice for the management of older people with dementia in the acute care setting: a review of the literature. Int J Older People Nurs.

[2] Sampson EL (2014). Behavioural and psychiatric symptoms in people with dementia admitted to the acute hospital: prospective cohort study. Br J Psychiatry.

[3] Lyketsos CG (2000). Mental and behavioral disturbances in dementia: findings from the cache county study on memory in aging. Am J Psychiatry.

[4] Banerjee S (2010). Living well with dementia—development of the national dementia strategy for England. Int J Geriatr Psychiatry.

[5] Freeman C, Joska J (2012). Management of behavioural and psychological symptoms of dementia. Contin Med Educ.

[6] Kales HC, Gitlin LN, Lyketsos CG (2014). Management of neuropsychiatric symptoms of dementia in clinical settings: recommendations from a multidisciplinary expert panel. J Am Geriatr Soc.

[7] Caramelli P, Mansur LL, Nitrini R, Stemmer B, Whitaker HA (1998). Chapter 32—language and communication disorders in dementia of the alzheimer type. Handbook of neurolinguistics.

[8] Potkins D (2003). Language impairment in dementia: impact on symptoms and care needs in residential homes. Int J Geriatr Psychiatry.

[9] Dewing J, Dijk S (2016). What is the current state of care for older people with dementia in general hospitals? A literature review. Dementia.

[10] Sampson EL (2015). Pain, agitation, and behavioural problems in people with dementia admitted to general hospital wards: a longitudinal cohort study. Pain.

[11] Hessler JB (2018). Behavioural and psychological symptoms in general hospital patients with dementia, distress for nursing staff and complications in care: results of the General Hospital Study. Epidemiol Psychiatr Sci.

[12] Galvin JE (2010). “Dementia-friendly hospitals: care not crisis”: an educational program designed to improve the care of the hospitalized patient with dementia. Alzheimer Dis Assoc Disord.

[13] Elvish R (2014). “Getting to Know Me”: the development and evaluation of a training programme for enhancing skills in the care of people with dementia in general hospital settings. Aging Ment Health.

[14] Prince M (2016). Recent global trends in the prevalence and incidence of dementia, and survival with dementia. Alzheimers Res Ther.

[15] Zekry D (2008). Demented versus non-demented very old inpatients: the same comorbidities but poorer functional and nutritional status. Age Ageing.

[16] Scerri A, Innes A, Scerri C (2017). Dementia training programmes for staff working in general hospital settings—a systematic review of the literature. Aging Ment Health.

[17] Zygouris S, Tsolaki M, Bamidis PD (2015). New technologies and neuropsychological evaluation of older adults: issues and challenges. Handbook of research on innovations in the diagnosis and treatment of dementia.

[18] Australian Government (2015) National framework for action on dementia 2015–2019. D.o.S. Services

[19] Department of Health (2009). Living well with dementia: a national dementia strategy.

[20] Wild K (2008). The status of computerized cognitive testing in aging: A systematic review. Alzheimers Dement.

[21] Zygouris S, Tsolaki M (2015). Computerized cognitive testing for older adults: a review. Am. J. Alzheimers Dis. Other. Demen.

[22] Tierney MC, Lermer MA (2010). Computerized cognitive assessment in primary care to identify patients with suspected cognitive impairment. J. Alzheimers Dis..

[23] Millett G (2018). Computerized cognitive testing in primary care: a qualitative study. Alzheimer Dis Assoc Disord.

[24] Tierney MC (2017). The effects of computerized cognitive testing of older patients on primary care physicians’ approaches to care: a Canadian study. Alzheimer Dis Assoc Disord.

[25] Robillard JM (2018). Patient perspectives of the experience of a computerized cognitive assessment in a clinical setting. Alzheimers Dement.

[26] Kipturgo MK (2014). Attitudes of nursing staff towards computerisation: a case of two hospitals in Nairobi, Kenya. BMC Med Inform Decis Mak.

[27] Kaye SP (2017). Nurses’ attitudes toward meaningful use technologies: an integrative review. Comput Inform Nurs..

[28] Huryk LA (2010). Factors influencing nurses’ attitudes towards healthcare information technology. J Nurs Manag.

[29] Ifinedo P (2018). Empirical study of nova scotia nurses’ adoption of Healthcare information systems: implications for management and policy-making. Int J Health Policy Manag.

[30] Safi S, Thiessen T, Schmailzl KJG (2018). Acceptance and resistance of new digital technologies in medicine: qualitative study. JMIR Res Protoc.

[31] Ifinedo P (2016). The moderating effects of demographic and individual characteristics on nurses’ acceptance of information systems: a canadian study. Med Inf.

[32] Kuo K-M, Liu C-F, Ma C-C (2013). An investigation of the effect of nurses’ technology readiness on the acceptance of mobile electronic medical record systems. BMC Med Inform Decis Mak.

