# Supplementary material for: Views of nursing staff on computerized dementia screening: A validation and pilot study in a general hospital
Source: Z Gerontol Geriatr. 2019 Oct 22;52(Suppl 4):258–63. doi: 10.1007/s00391-019-01633-0 (PMC6821656; doi:10.1007/s00391-019-01633-0)
Supplement: Supplementary file 1 — Questionnaire for the use of new technologies for timely detection of dementia [file 391_2019_1633_MOESM1_ESM.docx]

| **Questionnaire for the use of new technologies for timely detection of dementia** |
| --- |

| **1. Do you know that there are tests for the detection of dementia that are administered through a PC or tablet device?** | |
| --- | --- |
|  | О Yes  О No |

| **2. How desirable do you consider the following characteristics of computerized dementia screening tests?** | | | | | |
| --- | --- | --- | --- | --- | --- |
|  | ***Not at all*** | ***A little*** | ***So-so*** | ***A lot*** | ***Very much*** |
| **Brief administration** |  |  |  |  |  |
| **Ability to be administered by most members of staff (nurses/ doctors/ psychologists/ other healthcare personnel)** |  |  |  |  |  |
| **Automated administration and scoring** |  |  |  |  |  |
| **Embedded diagnostic algorithm** |  |  |  |  |  |
| **Provision of information for care/ referral after the end of the examination** |  |  |  |  |  |
| **Other: ……………………………………………….. ……………………………………………….. ……………………………………………….. ………………………………………………..** |  |  |  |  |  |

| **3. How much time could you devote to be trained in the use of computerized dementia screening tests?** | |
| --- | --- |
|  | О Less than 4 hours  О 4 – 8 hours  О 1 – 2 days  О 3 – 4 days  О 1 week |

| **4. How much time could you devote (during your shift) to examine an older adult with a computerized dementia screening test?** | |
| --- | --- |
|  | О Less than 5 minutes  О 5 – 10 minutes  О 10 – 20 minutes  О 20 – 40 minutes  О 40 – 60 minutes |

| **5. How interested you would be in using tests that are administered to relatives of patients and to patients with a high level of functionality, and can be used autonomously by the examinee without your participation in the examination (self-administered tests)?** | | | | |
| --- | --- | --- | --- | --- |
| ***Not at all*** | ***A little*** | ***So-so*** | ***A lot*** | ***Very much*** |
|  |  |  |  |  |

| **6. Do you believe that older adults without serious memory issues and with good functionality would be interested in using a self-administered cognitive assessment test?** | | | | |
| --- | --- | --- | --- | --- |
| ***Not at all*** | ***A little*** | ***So-so*** | ***A lot*** | ***Very much*** |
|  |  |  |  |  |

| **7. Do you believe that relatives of older adult patients will be interested in using a self-administered computerized questionnaire where they will evaluate the everyday functionality of the patient before admission to hospital and receive information about the probability of the patient suffering from dementia?** | | | | |
| --- | --- | --- | --- | --- |
| ***Not at all*** | ***A little*** | ***So-so*** | ***A lot*** | ***Very much*** |

| **8. Which factors do you believe impede the integration of computerized screening tests in your hospital?** | |
| --- | --- |
|  | О Cost of equipment  О Cost of software  О Lack of training  О Lack of a plan for their integration in the daily routine of the hospital  О Time needed for staff training  О Time needed for their use |
